# The adhesion receptor GPR56 is activated by extracellular matrix collagen III to improve β-cell function

**DOI:** 10.1007/s00018-018-2846-4

**Published:** 2018-05-31

**Authors:** Oladapo E. Olaniru, Attilio Pingitore, Stefanie Giera, Xianhua Piao, Ramón Castañera González, Peter M. Jones, Shanta J. Persaud

**Affiliations:** 1 Department of Diabetes, School of Life Course Sciences, King’s College London, Guy’s Campus, London, SE1 1UL UK; 2 Division of Newborn Medicine, Department of Medicine, Boston Children’s Hospital and Harvard Medical School, Boston, MA 02115 USA; 3 Department of General Surgery, Rio Carrión Hospital, University Hospital Complex of Palencia, Palencia, Spain

**Keywords:** Islets, Collagen III, GPR56, Extracellular matrix, Insulin secretion, Calcium

## Abstract

**Aims:**

G-protein coupled receptor 56 (GPR56) is the most abundant islet-expressed G-protein coupled receptor, suggesting a potential role in islet function. This study evaluated islet expression of GPR56 and its endogenous ligand collagen III, and their effects on β-cell function.

**Methods:**

GPR56 and collagen III expression in mouse and human pancreas sections was determined by fluorescence immunohistochemistry. Effects of collagen III on β-cell proliferation, apoptosis, intracellular calcium ([Ca^2+^]_i_) and insulin secretion were determined by cellular BrdU incorporation, caspase 3/7 activities, microfluorimetry and radioimmunoassay, respectively. The role of GPR56 in islet vascularisation and innervation was evaluated by immunohistochemical staining for CD31 and TUJ1, respectively, in pancreases from wildtype (WT) and *Gpr56*^−/−^ mice, and the requirement of GPR56 for normal glucose homeostasis was determined by glucose tolerance tests in WT and *Gpr56*^−/−^ mice.

**Results:**

Immunostaining of mouse and human pancreases revealed that GPR56 was expressed by islet β-cells while collagen III was confined to the peri-islet basement membrane and islet capillaries. Collagen III protected β-cells from cytokine-induced apoptosis, triggered increases in [Ca^2+^]_*i*_ and potentiated glucose-induced insulin secretion from WT islets but not from *Gpr56*^−/−^ islets. Deletion of GPR56 did not affect glucose-induced insulin secretion in vitro and it did not impair glucose tolerance in adult mice. GPR56 was not required for normal islet vascularisation or innervation.

**Conclusion:**

We have demonstrated that collagen III improves islet function by increasing insulin secretion and protecting against apoptosis. Our data suggest that collagen III may be effective in optimising islet function to improve islet transplantation outcomes, and GPR56 may be a target for the treatment of type 2 diabetes.

**Electronic supplementary material:**

The online version of this article (10.1007/s00018-018-2846-4) contains supplementary material, which is available to authorized users.

## Introduction

The extracellular matrix (ECM) is a specialised structure formed from secreted molecules such as proteins, proteoglycans and polysaccharides. ECM provides mechanical support and anchorage for cells, and it also regulates cellular processes such as adhesion, differentiation and survival via interactions with cell surface receptors that mediate signal transduction from the extracellular environment to the cell interior [1, 2]. Islets of Langerhans have a complex three-dimensional anatomy [3], including a surrounding ECM capsule known as the peri-insular basement membrane (BM). In addition, deposits of ECM are produced within islets by vascular endothelial cells, forming the peri-vascular BM [4]. The islet BM is a dynamic complex of proteins that is continually modified throughout life and it is composed primarily of laminin and collagens [5]. Most, if not all, islet β-cells are in direct contact with the BM [6] and it is well established that interactions with the BM directly influence β-cell survival, proliferation and insulin secretion [7, 8]. Conversely, reduced islet-ECM interactions are associated with deleterious outcomes. For example, the peri-insular BM is one of the first structures to be compromised during the development of type 1 diabetes [9], and loss of peri-insular BM during islet isolation leads to increased islet cell apoptosis [10].

Many in vitro studies of islet-ECM interactions have used complex ECM matrices laid down by a variety of cell types [7, 8], although some information is also available about individual roles of the more common ECM components. For example, collagen IV, an abundant protein in islet ECM, promoted adhesion, migration and insulin secretion in studies using human β-cells maintained in culture [11]. Collagen III is a major structural component of the ECM of many tissues and it is present in the pancreas of various mammals, including rodents, pigs and humans [12]. It has been identified as a ligand for an adhesion class G-protein-coupled receptor (GPCR), GPR56 [13], suggesting that it has a signalling role in addition to its structural function. Structurally, adhesion GPCRs have an unusually long N-terminal domain with many sticky motifs, allowing them to mediate cell–cell and cell–matrix interactions [14].

GPR56 is highly expressed in regions of the CNS involved in regulating food intake and energy homeostasis [15, 16], and in metabolically active peripheral tissues such as skeletal muscle, liver and adipose [17, 18]. In peripheral tissues, GPR56 expression was reported to be highest in mouse pancreas [16], in keeping with our more recent observations that it is the most abundant islet GPCR [13, 19]. Although the function of islet GPR56 is not well understood, its expression is reduced in islets obtained from type 2 diabetes (T2D) donors [20, 21], consistent with a role in regulating β-cell function. In the current study, we have investigated whether collagen III exerts paracrine effects by activation of GPR56 to regulate β-cell function.

## Experimental procedures

### Materials

All general laboratory reagents and cell culture materials used in this study were obtained from Sigma-Aldrich (Dorset, UK) unless otherwise stated. Other materials used include: caspase-Glo 3/7 Assay kits from Promega (Southampton, UK); BrdU Cell Proliferation ELISA kits from Cell Signalling Technology (Leiden, The Netherlands); TNFα, IL-1β and IFNγ from PeproTech (London, UK); and recombinant, full length collagen III (Abcam ab73160) from Abcam (Cambridge, UK). Collagen III is provided commercially in liquid form, made soluble by the presence of 10 mM HCl. In our experiments using soluble collagen III 10 mM HCl was added to negative controls and all solutions were buffered to a physiological pH of 7.4 before experimental use.

### Animals and islets

GPR56 null (*Gpr56*^−/−^) mice and their wildtype (WT) littermates were bred and genotyped as previously described [22], and colonies were maintained at Boston Children’s Hospital with free access to food and water. Islets were isolated from Jcl:ICR mice, and from G*pr56*^−/−^ and age-matched WT mice by collagenase digestion of the pancreas [23] and maintained in culture overnight at 37 °C in RPMI medium supplemented with 10% FBS and 100U/ml penicillin/0.1 mg/ml streptomycin prior to functional studies. The Ethics Committee of King’s College London or the Animal Use and Care Committee of Boston Children’s Hospital approved all animal protocols. Mouse insulinoma β-cells (MIN6) were maintained in monolayer culture as previously described [24]. Human islets and human exocrine cells were isolated from non-diabetic donor pancreases at King’s College Hospital, London, with ethical approval.

### Reverse transcription PCR

Total RNAs from MIN6 β-cells, mouse islets, mouse exocrine cells, human islets and human exocrine cells were extracted and reverse transcribed to cDNAs, which were then amplified by standard PCR using primers specific for mouse *Col3a1* (sense 5′-GTCCACGAGGTGACAAAGGT-3′, antisense 5′-GATGCCCACTTGTTCCATCT-3′), human *COL3A1* (sense 5′-GACCCTAACCAAGGATGCAA-3′, antisense 5′-GGAAGTTCAGGATTGCCGTA-3′), mouse *Actb* (sense 5′-AGCCATGTACGTAGCCATCC-3′, antisense 5′-TCTCAGCTGTGGTGGTGAAG-3′) and human *ACTB* (sense 5′-CGTGCGTGACATTAAGGAGA-3′, antisense 5′-CAGGCAGCTCGTAGCTCTTC-3′).

### Immunohistochemistry

Wax-embedded freshly retrieved mouse pancreases and non-diabetic and T2D human pancreas blocks were cut to 5 μm thick sections, de-waxed and antigenicity was restored by heat-induced epitope retrieval. Sections were blocked for 1 h (1% BSA, 10% normal goat serum, 0.1% triton-X-100 in PBS) then incubated overnight with appropriate primary antibodies. After washing in PBS, pancreas sections were incubated with fluorophore-conjugated secondary antibodies for 1 h followed by nuclei counterstaining with DAPI (1:2000). Images were captured using Nikon Eclipse Ti or Nikon TE 2000-U inverted microscopes and quantified using Image J software. Islet nerve and vascular areas were determined as area of TUJ1 and CD31 immunostaining, respectively, within insulin-positive islet cells. Fluorescently labelled individual endothelial cells or cell clusters, that were clearly distinct from adjacent cells, were counted as a single blood vessel according to the Weidner method [25]. The primary and secondary antibodies used and their dilutions are listed in Supplementary Table 1.

### Insulin secretion and content

Isolated mouse islets were incubated in a physiological salt solution [26] in the absence or presence of 100 nM soluble recombinant collagen III for 1 h. In parallel experiments to assess the chronic effect of collagen III on insulin secretion, islets were cultured for 48 h on dishes coated with 100 nM collagen III before being retrieved and exposed to either 2 or 20 mM glucose. For the dynamic insulin secretion experiments, groups of 40 islets were perifused at 37 °C and samples were collected every 2 min [27]. Insulin secretion in the static incubation and perifusion experiments was quantified by radioimmunoassay [28]. To measure insulin content, groups of 10 size-matched islets from adult *Gpr56*^−/−^ and WT mice were incubated in 200 μl of acidified ethanol overnight at 4 °C to extract insulin and kept at − 20 °C until insulin was quantified by radioimmunoassay.

### Apoptosis and proliferation

Groups of 20,000 MIN6 β-cells were maintained in culture in DMEM containing 2% FBS in the absence or presence of 100 nM soluble collagen III for 48 h, with a subsequent 20 h incubation in the absence or presence of a cytokine cocktail to induce apoptosis (1U/µl TNFα, 0.05U/µl IL-1β and 1U/µl IFNγ). Apoptosis was determined by measuring caspase 3/7 activities as described previously [29]. Groups of 20,000 MIN6 β-cells were incubated in the absence or presence of soluble collagen III for 48 h before being labelled with 100 µM BrdU for 4 h at 37 °C, and β-cell proliferation was determined by colorimetric quantification of BrdU incorporation into cellular DNA [24].

### Single cell calcium microfluorimetry

MIN6 β-cells were seeded at a density of 100,000 cells on acid–ethanol washed glass coverslips and maintained overnight at 37 °C in DMEM. The cells were incubated with 5 µM of the calcium fluorophore Fura-2AM for 30 min before being perifused with physiological solution containing 2 mM or 20 mM glucose in the absence or presence of soluble collagen III. Cells were alternately exposed to light at 340 nm and 380 nm and emission was filtered at 510 nm to determine changes in intracellular calcium ([Ca^2+^]_*i*_) [30].

### Glucose tolerance tests

Glucose tolerance was measured in *Gpr56*^−/−^ mice and age-matched WT littermates by intraperitoneal administration of 2 g glucose/kg body weight after an overnight fast [31]. Plasma glucose levels were measured prior to glucose administration and at 15, 30, 60, 90, and 120 min post injection using a glucometer.

### Statistical analyses

Normally distributed data were analysed using two-tailed Student’s *t* tests or ANOVA, as appropriate and the Mann–Whitney *U* test was used where data did not follow Gaussian distribution. Repeated measurements in the same animal at different time points during glucose tolerance tests were determined by two-way repeated measurement ANOVA with Bonferroni’s post hoc tests. For histological analyses, images were scored blindly before quantification.

## Results

### Expression of collagen III and GPR56 in mouse and human islets

**Fig. 1:**
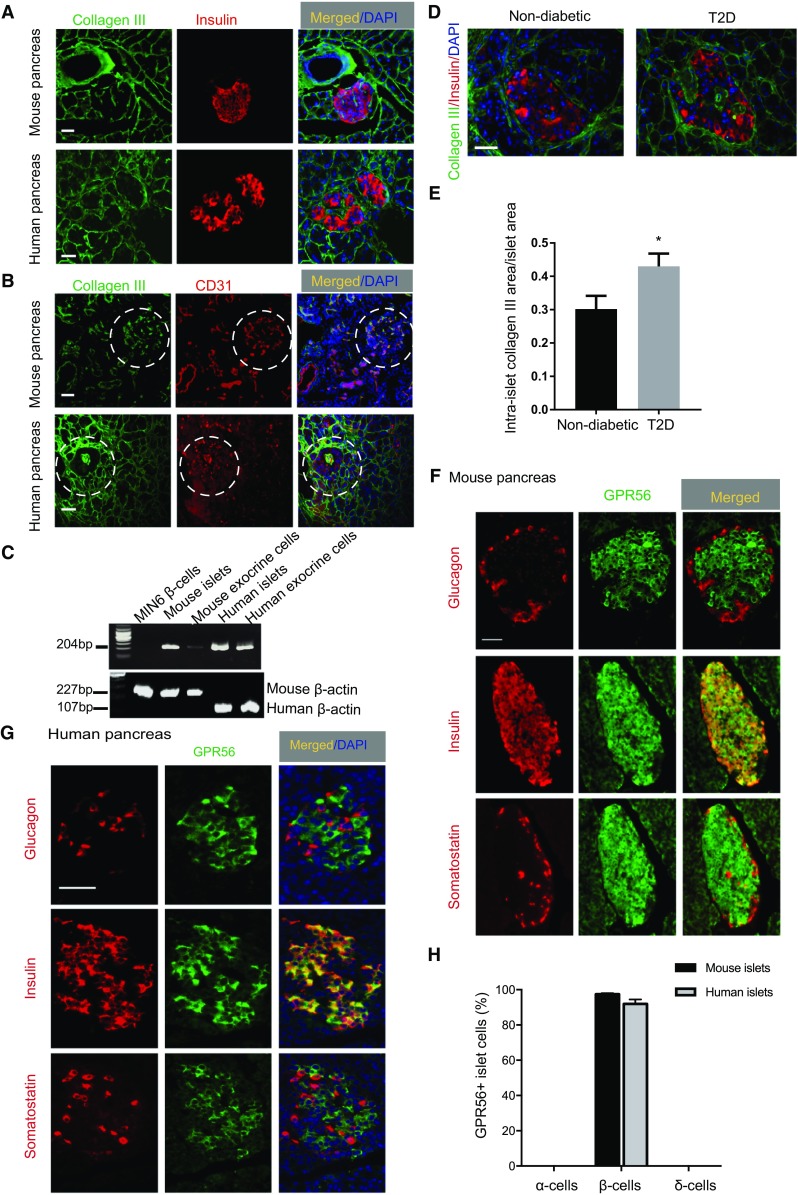
Expression of collagen III and GPR56 in mouse and human islets. **a** Mouse and human pancreas sections were immunoprobed with antibodies directed against collagen III (green) and insulin (red). **b** Mouse and human pancreas sections were immunoprobed with antibodies directed against collagen III (green) and CD31 (red). Islets are identified by dotted circles. **c** Products of RT-PCR amplification using mouse and human *col3a1* primers (upper panel) and β-actin (lower panel) with cDNAs obtained from MIN6 β-cells, mouse and human islets and exocrine cells. Amplicons correspond to the predicted sizes of the nucleotide sequence generated using the chosen mouse and human primer pairs. **d** Non-diabetic and T2D human pancreas sections were immunoprobed with antibodies directed against collagen III (green) and insulin (red). **e** The area of intra-islet collagen III immunoreactivity is represented as a fraction of the islet area. Data are presented as mean + SEM, *n* = 3 sections, 24 non-diabetic islets and 37 T2D islets. *p < 0.05. **f** Mouse pancreas sections were immunoprobed with antibodies directed against GPR56 (green) and insulin, glucagon and somatostatin (red). **g** Human pancreas sections were immunoprobed with antibodies directed against GPR56 (green) and insulin, glucagon and somatostatin (red). **h** The number of individual mouse and human islet cells expressing GPR56 is represented as a percentage of the total number of islet cells. Data are represented as mean + SEM, *n* = 5 sections, 289 human islet cells and 113 mouse islet cells. Scale bar for **a**, **b**, **d**, **f**, and **g** = 50 μm

Fluorescence immunohistochemical analysis of mouse pancreas sections revealed the presence of collagen III immunostaining around the lobar and acinar septa of the pancreas, at the peri-islet BM and within islets, as shown in Fig. 1a. Collagen III did not co-localise with insulin in mouse and human islets, suggesting that it was not synthesised within β-cells (Fig. 1a), but it was co-expressed by cells that were immuno-positive for the vascular endothelial marker CD31 (Fig. 1b). Consistent with this, collagen III mRNA was not detected in MIN6 β-cells although it was present in mouse and human islets (Fig. 1c). Amplification of MIN6 β-cell β-actin (Fig. 1c) indicated that the absence of a product with collagen III primers was not a consequence of poor quality MIN6 cell cDNA. Given that ~ 60% of islet endothelial cells are preserved after 24 h maintenance of islets in culture [32] and RNAs used for RT-PCR were extracted from islets following overnight culture after isolation, it is likely that identification of collagen III mRNA in islet samples (Fig. 1c) and its co-expression with CD31 (Fig. 1b) reflects its expression by islet vascular endothelial cells. Immunohistochemical analysis of collagen III expression in T2D human pancreas sections revealed that there was a significant increase in collagen III deposition within the islets compared to non-diabetic pancreas (Fig. 1d, e).

Immunostaining of mouse (Fig. 1f) or human (Fig. 1g) pancreas sections for GPR56 demonstrated that it was highly expressed within islets, with much lower expression in the surrounding exocrine tissue. GPR56 immunoreactivity was not localised exclusively at the plasma membrane, most likely as a consequence of the membrane permeabilisation protocol that was used to allow dual immunostaining with islet hormones. GPR56 localised to the cytoplasm in Fig. 1f, g may reflect newly synthesised GPR56 being trafficked to the plasma membrane or GPR56 undergoing internalisation and/or recycling to the plasma membrane, a common feature of GPCRs. Dual immunostaining for insulin, glucagon or somatostatin demonstrated that GPR56 was exclusively expressed by β-cells in both mouse and human islets and Fig. 1h shows that > 95% of β-cells in both mouse and human islets were immunoreactive for GPR56.

### Effects of collagen lll on β-cell function

**Fig. 2:**
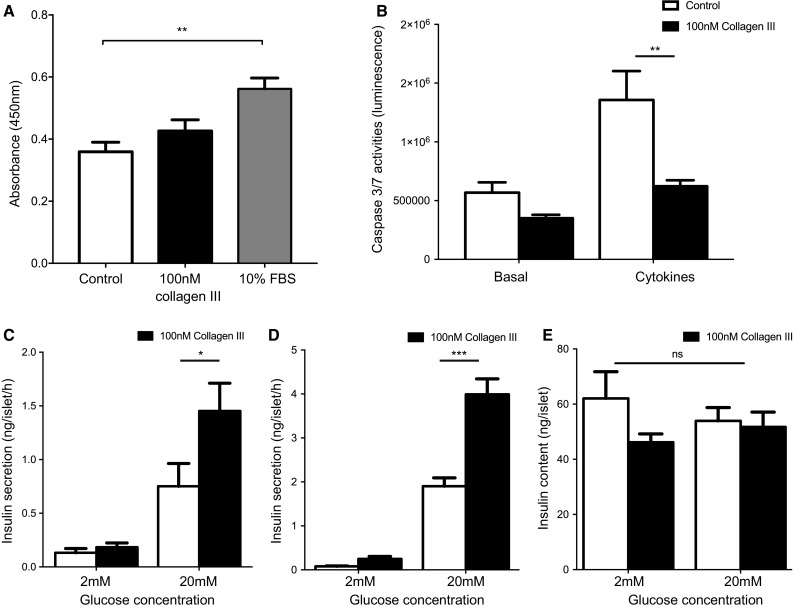
Effects of collagen III on β-cell function. **a** Proliferating MIN6 β-cells labelled with BrdU were determined by quantification of absorbance at 450 nm. Data are mean + SEM, *n* = 4 independent experiments, **p < 0.01, one-way ANOVA. **b** Caspase 3/7 activities of MIN6 β-cells were measured by a luminescence-based method. Data are mean + SEM, *n* = 5, **p < 0.01. **c** Insulin secretion from mouse islets after a 1 h static incubation was quantified by radioimmunoassay. Data are mean + SEM, *n* = 3 independent experiments, *p < 0.05. **d** Mouse islets that had been maintained in culture on Petri dishes coated with vehicle (10 mM HCl) or 100 nM collagen III for 48 h were then incubated for 1 h in the presence of 2 or 20 mM glucose and insulin secretion was quantified by radioimmunoassay. Data are mean + SEM, *n* = 6–8 replicates from 5 mice, ***p < 0.001. **e** Insulin content of groups of 10 islets from the treatment groups in **d** was determined by radioimmunoassay. Data are mean + SEM, ns = not significant. The p values in **b**–**e** were calculated using two-way ANOVA with Bonferroni’s multiple comparison test

GPR56 expression within islets suggested a β-cell-specific function for this receptor so we measured the effects of its endogenous agonist, collagen lll, on β-cell proliferation, survival and insulin secretion in vitro. Pre-treatment of MIN6 β-cells with 100 nM soluble collagen lll for 48 h had no detectable effects on proliferation, as assessed by BrdU incorporation, whereas 10% FBS produced the expected increase in proliferation (Fig. 2a). However, pre-treatment of MIN6 β-cells with 100 nM soluble collagen lll significantly reduced apoptosis induced by a cocktail of cytokines, without having significant effect on basal rates of apoptosis (Fig. 2b).

The acute effects of collagen III on insulin secretion were investigated by incubating mouse islets for 1 h at sub-stimulatory (2 mM) or maximal stimulatory (20 mM) concentrations of glucose in the absence or presence of 100 nM soluble collagen III. Figure 2c shows that this acute exposure to soluble collagen III had no effect on basal insulin secretion at 2 mM glucose but it caused a significant potentiation of glucose-induced insulin secretion, consistent with the activation of a stimulatory GPCR. The chronic effects of collagen III on insulin secretion were assessed using islets which were pre-cultured for 48 h on a layer of collagen III to mimic the ECM environment, followed by an acute (60 min) exposure to 2 mM or 20 mM glucose in the absence of collagen lll. Pre-treatment with collagen lll had no effect on basal insulin secretion, but significantly potentiated glucose-induced insulin secretion, as shown in Fig. 2d. This enhanced glucose-induced insulin secretion was not caused by increased islet insulin content because there were no significant differences in insulin content of islets that were pre-treated for 48 h with collagen III in the presence of either 2 or 20 mM glucose (Fig. 2e).

### Collagen III stimulates insulin secretion via increases in intracellular calcium

**Fig. 3:**
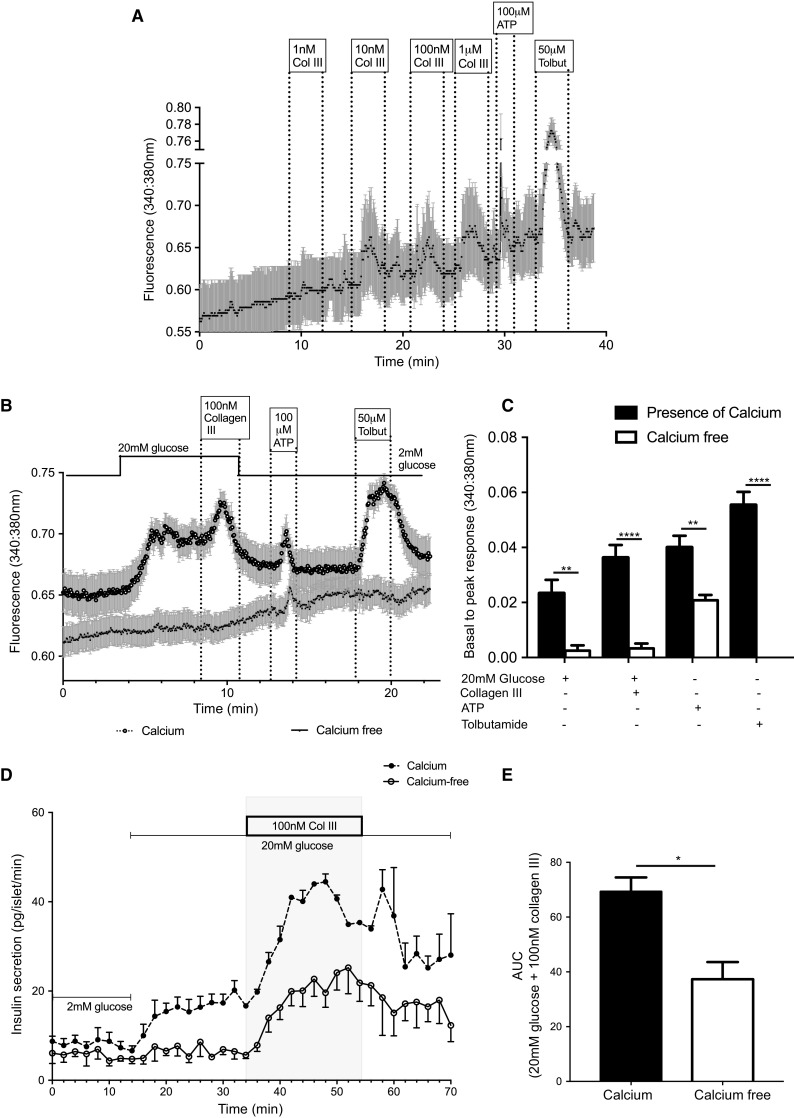
Collagen III increases intracellular Ca^2+^ in β-cells. **a** MIN6 β-cells loaded with Fura-2 were perifused with physiological solution supplemented with different concentrations of collagen III at the indicated time points and changes in intracellular calcium are expressed as 340/380 nm ratiometric data. Data are mean ± SEM, *n* = 19 cells, representative of three separate experiments. **b** MIN6 β-cells loaded with Fura-2 were perifused with either calcium-free physiological buffer in the presence of 1 mM EGTA or buffer containing 2 mM calcium, and supplemented with agonists as shown. Data are mean + SEM, *n* = 24 cells, representative of three separate experiments. **c** Peak amplitude responses of MIN6 β-cells to the agonists shown in **b** in the presence or absence of extracellular calcium. **p < 0.01, ****p < 0.0001, Two-way ANOVA, with Tukey’s posthoc test. **d** Mouse islets were perifused with either calcium-free physiological buffer or buffer containing 2 mM calcium, supplemented as shown, and insulin secretion was quantified by radioimmunoassay. Data are mean ± SEM, *n* = 4 replicates of 40 islets each isolated from 5 mice. **e** Area under the curve quantification of collagen III-induced insulin secretion shown in **d** between 34 and 54 min in the presence or absence of extracellular calcium. Data are mean + SEM, *p < 0.05, unpaired *t* test

Calcium-induced exocytosis of insulin granules is the primary determinant of stimulated insulin secretion [33], so we assessed the effects of collagen III on β-cell intracellular Ca^2+^ ([Ca^2+^]_*i*_) by live cell imaging using Fura-2-loaded MIN6 β-cells. The traces in Fig. 3a, b show real-time changes in [Ca^2+^]_*i*_ in response to increasing concentrations of soluble collagen lll in the presence of 2 mM glucose (A) or 20 mM glucose (B). At concentrations of 10 nM and above collagen lll caused rapid and reversible increases in basal [Ca^2+^]_*i*_, similar to those induced by ATP, another GPCR ligand that acts through purinergic receptors (Fig. 3a). Soluble collagen III also augmented the glucose-induced increase in [Ca^2+^]_*i*_, as shown in Fig. 3b. Thus, 20 mM glucose elicited a rapid and prolonged increase in [Ca^2+^]_*i*_ in MIN6 β-cells, which was further enhanced by the presence of 100 nM soluble collagen lll to produce an additional and reversible increase in basal to peak ratio of [Ca^2+^]_*i*_. In the absence of extracellular calcium and the presence of the calcium chelator, EGTA (1 mM), neither 20 mM glucose nor 100 nM collagen III increased [Ca^2+^]_*i*_ (Fig. 3b, c), indicating that an influx of extracellular calcium was the primary source of elevated [Ca^2+^]_*i*_ in response to these agents. Under the same conditions, 100 µM ATP continued to increase [Ca^2+^]_*i*_, consistent with its ability as a purinergic receptor agonist to mobilise Ca^2+^ from internal stores [34]. Tolbutamide is a sulphonylurea that closes K_ATP_ channels, with subsequent depolarisation of the plasma membrane leading to calcium influx through voltage-gated calcium channels [35]. It cannot elevate [Ca^2+^]_*i*_ without calcium influx as shown by the complete loss of its effects on [Ca^2+^]_*i*_ when extracellular calcium was removed from the physiological buffer (Fig. 3b, c).

Stimulation of insulin secretion by soluble collagen lll was partially dependent on an influx of extracellular calcium, as shown in Fig. 3d. Measurement of dynamic insulin secretion from mouse islets in a perfusion system demonstrated a complete inhibition of insulin secretion in response to 20 mM glucose in the absence of extracellular calcium. Potentiation of the glucose-induced secretory response by 100 nM soluble collagen lll was significantly reduced in the absence of extracellular calcium although it was still able to stimulate insulin secretion under the calcium-free conditions (Fig. 3d, e).

### Collagen III stimulates insulin secretion via GPR56 activation

**Fig. 4:**
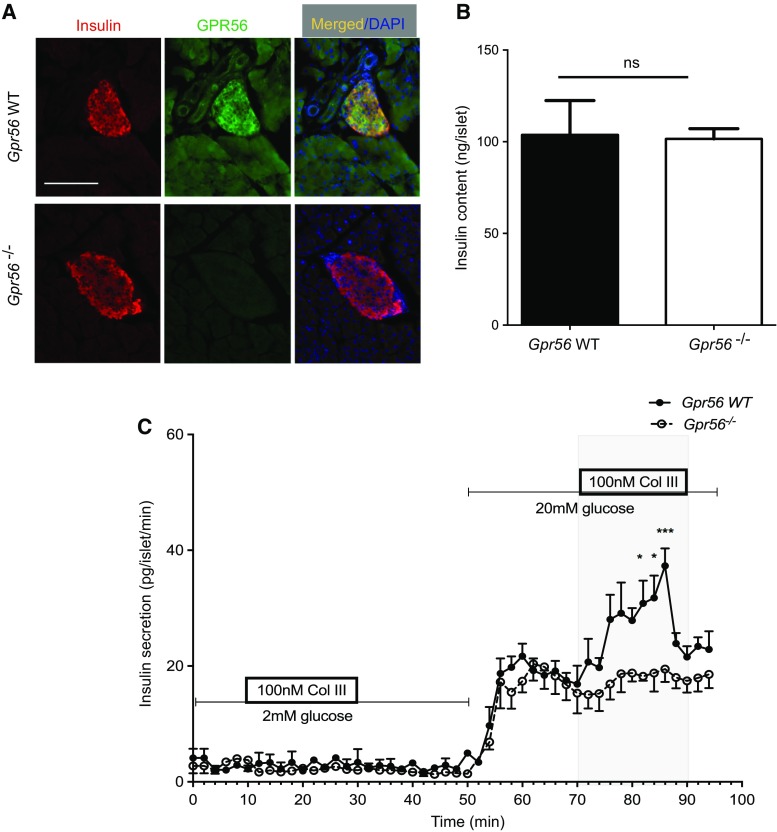
Collagen III stimulates insulin secretion via GPR56. **a** Pancreas sections from adult WT and *Gpr56*^−/−^ mice were immunoprobed with antibodies directed against GPR56 (green) and insulin (red). Scale bar = 50 μm. **b** Insulin content of islets isolated from WT and *Gpr56*^−/−^ mice was determined by radioimmunoassay. Data are mean + SEM, *n* = 3 batches of 10 islets pooled from 5 mice/genotype, *ns* not significant. **c** Islets isolated from WT and *Gpr56*^−/−^ mice were perifused with supplements as shown and insulin secretion was determined by radioimmunoassay. Data are mean ± SEM, *n* = 4 replicates of 40 islets each isolated from 5 mice per genotype, *p < 0.05, ***p < 0.001, two-way ANOVA, with Bonferroni’s multiple comparison test

To investigate whether collagen III stimulates insulin secretion by activating GPR56 we used islets isolated from *Gpr56*^−/−^ mice and their WT littermates. Fluorescence immunohistochemistry demonstrated that GPR56 immunoreactivity was not detectable in the β-cells in *Gpr56*^−/−^ pancreas sections, although it was readily detectable in WT β-cells (Fig. 4a). There was no significant difference in the insulin content of islets isolated from WT or *Gpr56*^−/−^ mice (Fig. 4b). However, the previously observed stimulatory effects of soluble collagen lll on insulin secretion were abolished in the *Gpr56*^−/−^ islets such that the collagen lll-dependent potentiation of glucose-induced insulin secretion observed in WT islets was completely absent in the islets from *Gpr56*^−/−^ mice (Fig. 4c). It can also be seen from Fig. 4c that the biphasic insulin secretory response to 20 mM glucose was similar in islets from WT and *Gpr56*^−/−^ mice, suggesting that GPR56 activity is not required for β-cell stimulus-secretion coupling in response to glucose.

### GPR56 is not required for islet innervation and vascularisation

**Fig. 5:**
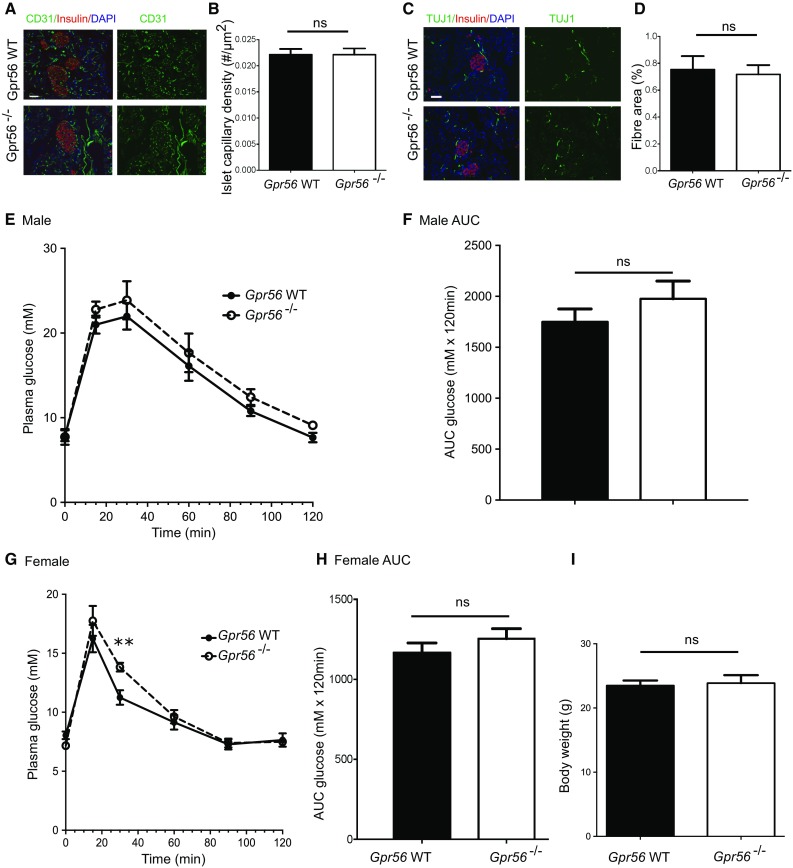
Effects of GPR56 deletion in vivo. **a** Pancreas sections from P9 WT and *Gpr56*^−/−^ mice were immunoprobed with antibodies directed against CD31 (green) and insulin (red). **b** Islet capillary density was quantified by determining the number of CD31^+^ blood vessels within the insulin^+^ islet area in the pancreas sections from WT and *Gpr56*^−/−^ mice. Data are mean + SEM of *n* = 10 sections. **c** Pancreas sections from P9 WT and *Gpr56*^−/−^ mice were immunoprobed with antibodies directed against TUJ1 (green) and insulin (red). Scale bar = 50 μm. **d** Nerve fibre density was quantified by determining the number of TUJ1^+^ neurons within the insulin^+^ islet area in the pancreas sections from WT and *Gpr56*^−/−^ mice. Data are mean + SEM of *n* = 3 mice per genotype, ten sections per mouse. Scale bar for **a** and **c** = 50 μm. **e** Plasma glucose concentrations were measured at time 0, 15, 30, 60, 90 and 120 min post 2 g/kg glucose administration to 16 h fasted 8 week old male WT and *Gpr56*^−/−^ mice (weight, WT: 23.4 ± 1.1 g, *Gpr56*^−/−^: 23.6 ± 1.5 g). Data are mean ± SEM, *n* = 5 mice per genotype. **f** Total glucose area under the curve (AUC) over 120 min for the glucose tolerance tests in panel **e** was quantified for male WT and *Gpr56*^−/−^ mice. **g**, **h** Plasma glucose concentrations and AUC of 8 week old female WT and *Gpr56*^−/−^ mice (weight, WT: 20.9 ± 0.4 g, *Gpr56*^−/−^: 24.6 ± 1.3 g) were also determined at the indicated time points. Data are mean ± SEM, *n* = 4 female mice per genotype, **p < 0.01, unpaired *t* test. **i** Total body weight of male WT and *Gpr56*^−/−^ mice at 8 weeks. Data are mean + SEM of 7 WT and 8 *Gpr56*^−/−^ mice

Collagen III is the major ligand for GPR56 in the developing brain [36], and GPR56 has been implicated in angiogenesis by regulating expression of VEGF genes [37], so we used *Gpr56*^−/−^ mice to investigate a potential role for this receptor in islet innervation and vascularisation in early postnatal development. Pancreases from *Gpr56*^−/−^ or WT littermate mice at postnatal day 9 (P9) were immunostained for the vascular endothelial cell marker CD31 and for TUJ1, a neuron-specific structural protein [38]. As expected, CD31 immunoreactivity revealed that islets from both WT and *Gpr56*^−/−^ mice had a rich supply of blood vessels (Fig. 5a) and TUJ1 immunostaining indicated that islets were surrounded by nerve endings in WT and *Gpr56*^−/−^ mice (Fig. 5c). Analysis of islet capillary density and nerve fibre area demonstrated that there were no detectable differences between the WT and *Gpr56*^−/−^ mice (Fig. 5b, d), indicating that GPR56 is not required for appropriate vascularisation and innervation of islets.

### GPR56 deletion has little effect on glucose tolerance

Global deletion of GPR56 had relatively little effect on whole body fuel homeostasis in adult mice, as shown in Fig. 5e–h. There was no difference in the body weight of 8 week old *Gpr56*^−/−^ and WT mice maintained on a standard chow diet (Fig. 5i), indicating that GPR56 does not play a role in the regulation of total body mass under normal, non-stressed conditions. Although not statistically significant, glucose tolerance in *Gpr56*^−/−^ male mice showed higher serum glucose level at all time points (Fig. 5e), but we detected no genotype-dependent differences in the area under the curve (AUC) glucose levels calculated over the entire 120 min period (Fig. 5f). A similar trend was also observed in *Gpr56*^−/−^ female mice, with a significantly higher concentration of plasma glucose at 30 min post-injection (Fig. 5g). However, AUC calculations also did not show significant genotype-specific differences in glucose tolerance in the female mice (Fig. 5h).

## Discussion

There is a substantial body of evidence that β-cell function can be modified, normally in a beneficial manner, by direct contact with ECMs secreted by a variety of cell types [39, 40], or by individual molecular components of ECM [8, 41]. The canonical signalling mechanism from ECM to the β-cell intracellular compartment is via plasma membrane-spanning integrins which bind to protein elements of ECM, often collagen molecules, and transduce the signals inside β-cells via interactions with focal adhesion kinases [11, 42]. The majority of mechanistic studies on interactions between ECM or collagen and β-cells have largely focused on collagen-integrin interactions [11, 43] with little emphasis on GPCRs, even though the GPCR superfamily comprises the largest group of mammalian cell surface receptors [44]. Here, we demonstrate that collagen III is an abundant component of islet ECM found in both peri-islet and peri-vascular BMs and that it influences islet function via the activation of GPR56, as confirmed by the use of a mouse model in which GPR56 was deleted. Activation of β-cell GPR56 has several potentially beneficial effects, including the Ca^2+^-dependent potentiation of insulin secretion and protection against the deleterious effects of inflammatory cytokines. However, our observations also demonstrated that GPR56 activation does not influence other β-cell functions such as glucose sensing and cell proliferation, nor developmental processes such as vascularisation or innervation, which are regulated by ECM, integrins and/or GPR56 in other tissues [14, 45]. These observations suggest that collagen lll/GPR56 signalling complements the ECM/integrin pathway in β-cells, and is an important mechanism through which ECM can influence β-cell function.

Our data confirm earlier observations that collagen lll stimulates insulin secretion via β-cell GPR56 [21]. However, in contrast to observations that GPR56 down-regulation by sh/siRNAs reduces glucose-induced insulin secretion and insulin content [21], our measurements in islets isolated from *Gpr56*^−/−^ and WT littermate mice demonstrate that GPR56 is not required for glucose-induced insulin secretion, nor for the maintenance of insulin content. The reasons for these conflicting observations are unclear. However, it is possible that acute shRNA-mediated islet GPR56 down-regulation in vitro can impair insulin secretion but this is not seen in islets isolated from mice where GPR56 is deleted in utero due to undefined compensatory mechanisms in vivo.

Soluble collagen lll increased β-cell [Ca^2+^]_*i*_ in the presence of 2 mM glucose, but did not stimulate insulin secretion at this basal glucose concentration. This potentiating rather than initiating effect is commonly seen with Gq-coupled receptor agonists such as acetylcholine analogues [46] and indicates that elevation in [Ca^2+^]_*i*_ in the absence of stimulatory glucose concentrations is not sufficient to induce stimulation of insulin secretion. The effects of removing extracellular Ca^2+^ on collagen lll-induced increases in β-cell [Ca^2+^]_*i*_ imply an important role for changes in [Ca^2+^]_*i*_ in response to GPR56 activation, consistent with reports that GPR56 is coupled via both Gq and G_12/13_, pathways that are associated with elevations in [Ca^2+^]_*i*_ [18, 47]. Soluble collagen III did not increase [Ca^2+^]_*i*_ in the absence of extracellular calcium but it did significantly potentiate glucose-stimulated insulin release, albeit to a lesser extent than when islets were perifused with physiological solution containing 2 mM calcium. These data suggest that collagen III acts via a different cascade to promote insulin release in the absence of calcium influx. One possibility for this calcium influx-independent stimulatory effect of collagen III on insulin secretion is increased actin cytoskeleton re-modelling downstream of GPR56-mediated G_12/13_ signalling [48]. Alternatively, or additionally, GPR56 could be signalling in β-cells via cAMP elevations and activation of protein kinase A, which has been reported previously [21], although direct Gs-mediated coupling of GPR56 has not yet been identified.

We observed beneficial effects of GPR56 activation on insulin secretion after acute exposure to soluble collagen IIl, as used in a previous study [21], but also after more chronic exposure when the collagen III was configured as an adherent layer on a tissue culture plastic substrate to better mimic the interaction between the insoluble ECM and β-cells within the islet. These observations suggest that the collagen III in the peri-islet and peri-vascular BM, which is in close contact with the β-cells, will be able to exert a beneficial influence on β-cell function through activation of GPR56. Conversely, reduced collagen lll/GPR56 interactions would be predicted to result in impaired insulin secretion and increased susceptibility of β-cells to the deleterious effects of inflammatory cytokines. Our immunohistochemical data analysis showing that collagen III deposition was elevated within T2D islets is consistent with a recent report of increased collagen in islets of T2D pancreases, detected by picrosirius red staining [49]. Indeed, chronically high levels of collagen III in T2D may be responsible for the reduced Gpr56 expression observed in islets of individuals with T2D [21]. Since the ECM is not a fixed entity in situ [5], it is likely that ECM re-modelling and conformational changes allow variable interactions between collagen III and GPR56, such that GPR56 is not continually activated, and in this way collagen III can play a physiological signalling role in the regulation of β-cell function.

Our in vivo studies using *Gpr56*^−/−^ mice suggest that, under normal physiological conditions, GPR56 is not required for whole body glucose homeostasis in adult mice. This is consistent with the lack of detectable effects of GPR56 deletion on glucose-induced insulin secretion from isolated islets and might suggest that collagen lll/GPR56 interactions are more important in β-cell adaptations to metabolic stress, as has been reported for other GPCRs. For example, gene deletion studies have shown that GPR39 expression is not required for normal fuel homeostasis, nor for glucose-induced insulin secretion from isolated islets [50, 51], but *Gpr39*^−/−^ mice maintained on high fat or high sucrose diets become glucose intolerant due to reduced insulin secretion [52]. Further studies using *Gpr56*^−/−^ mice are required to define the role of GPR56 in β-cell responses to metabolic stressors, such as intake of diets high in fat or carbohydrate, where increased metabolic demand drives compensatory increases in β-cell mass and insulin output.

In summary, we have demonstrated that collagen III is a constituent of islet ECM, and that β-cells selectively express its receptor, GPR56. We have shown that collagen III influences β-cell function in terms of enhancing cell survival and insulin secretory responses, and that it acts via GPR56 to potentiate glucose-stimulated insulin secretion. Together, these data provide insights into the role played by the ECM protein collagen III and GPR56 in regulating islet function, which may be useful in optimising islet function prior to transplantation in type 1 diabetes patients, or in targeting GPR56 for new therapies for T2D.

## Electronic supplementary material

Below is the link to the electronic supplementary material.

Supplementary material 1 (DOCX 29 kb)
