# Supplementary material for: The adhesion receptor GPR56 is activated by extracellular matrix collagen III to improve β-cell function
Source: Cell Mol Life Sci. 2018 May 31;75(21):4007–19. doi: 10.1007/s00018-018-2846-4 (PMC6182347; doi:10.1007/s00018-018-2846-4)
Supplement: Supplementary file 1 — Supplementary material 1 (DOCX 29 kb) [file 18_2018_2846_MOESM1_ESM.docx]

**Supplementary Table 1. Antibodies for immunohistochemistry**

The primary and secondary antibodies were used for immunohistochemistry in mouse and human pancreas at the dilutions stated.

| **Primary antibodies** | Source | Dilution |
| --- | --- | --- |
| rabbit monoclonal anti-GPR56 (199)  IHC in Figure 4 | Piao’s lab | 1:250 |
| mouse polyclonal anti-GPR56  IHC in Figure 1 | Millipore; ABN16 | 1:25 |
| rabbit polyclonal anti-collagen III | Lifespan Biosciences; LS-B693 | 1:1000 |
| guinea-pig polyclonal anti-insulin | Dako; A0564 | 1:200 |
| mouse monoclonal anti-glucagon | Sigma; G2654 | 1:50 |
| Rat monoclonal anti-somatostatin | Abcam; ab30788 | 1:25 |
| rabbit monoclonal anti-TUJ1 | Covance; MRB-435 | 1:1000 |
| mouse monoclonal anti-CD31 | Life Technologies; MA-513188 | 1:50 |
| rat monoclonal anti-CD31 | BD Biosciences; 553370 | 1:100 |
| **Secondary antibodies** |  |  |
| Alexa-fluor 594 donkey anti-rat | Jackson Immunolab; 712-585-150 | 1:100 |
| Alexa-fluor 488 donkey anti-rabbit | Jackson Immunolab; 711-545-152 | 1:100 |
| Alexa-fluor 546 goat anti-guinea pig | Life Technologies; A11074 | 1:1000 |
| Alexa-fluor 488 goat anti-mouse | Life Technologies; A11029 | 1:1000 |
